# CircularRNA circ_0071269 knockdown protects against from diabetic cardiomyopathy injury by microRNA-145/gasdermin A axis

**DOI:** 10.1080/21655979.2021.2024688

**Published:** 2022-01-16

**Authors:** Lanfang Fu, Juyun Zhang, Zhu Lin, Yi Li, Guijun Qin

**Affiliations:** a Department of Endocrinology, The First Affiliated Hospital of Zhengzhou University, Zhengzhou City, China; b Department of Endocrinology, Haikou Hospital, Affiliated to Xiangya Medical College, Central South University, Haikou, China; c Department of Clinical Medicine, Hainan Medical College, Haikou, China

**Keywords:** circ_0071269, miR-145, GSDMA, diabetic cardiomyopathy, pyroptosis

## Abstract

Circular RNAs (circRNAs) are involved in the development and progression of diabetic cardiomyopathy (DCM). However, the specific function and underlying mechanism of circ_0071269 in DCM remains unclear. In our study, mRNA and miRNA expression was detected by real-time quantitative PCR (qRT-PCR). RNase R and actinomycin D treatment were applied to test the characteristics of circ_0071269. Cell Counting Kit-8 (CCK-8) assay, lactate dehydrogenase (LDH) and enzyme-linked immunosorbent assay (ELISA) kits were performed to determine the cell viability, cell LDH content and interleukin (IL)-1β and IL-18 levels, respectively. Cell death rate was determined by Flow cytometry, and Western blotting was for the protein expression levels. In addition, luciferase reporter and RNA pull-down assays were performed to confirm the binding relationship between miR-145 and circ_0071269 or gasdermin A (GSDMA). Echocardiography, Hematoxylin and Eosin (HE) Staining, and Immunohistochemical (IHC) Staining were performed to evaluate myocardial damage *in vivo*. We found that circ_0071269 was significantly overexpressed in H9c2 cells upon treatment with high glucose. Knockdown of circ_0071269 promoted cell viability and inhibited the inflammatory response, cytotoxicity, and pyroptosis of H9c2 cells *in vitro*. Moreover, circ_0071269 sponges miR-145 to upregulate GSDMA. A miR-145 inhibitor antagonized the effects of circ_0071269 knockdown on the cellular functions of H9c2 cells, while the effects of miR-145 were abrogated by the overexpression of GSDMA. Meanwhile, knockdown of circ_0071269 attenuated cardiac dysfunction of DM mice. Hence, circ_0071269 may promote the development of DCM through the miR-145/GSDMA axis and thus provide a novel marker for the treatment of DCM.

## GRAPHICAL ABSTRACT

**Figure:**
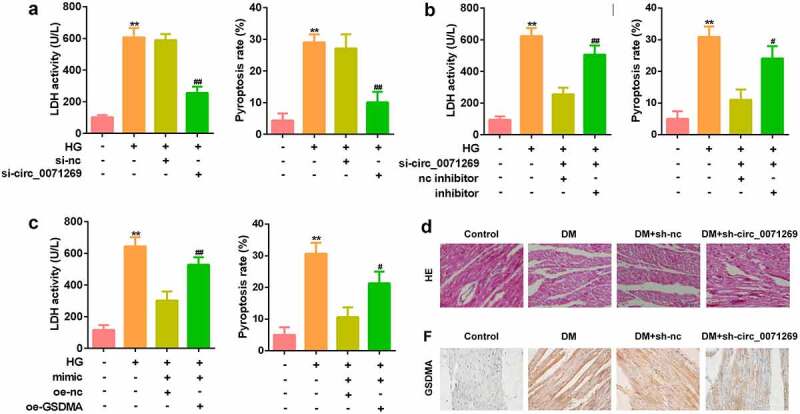

## Introduction

Diabetic cardiomyopathy (DCM) is a major complication of diabetes and a major cause of death in patients with chronic diabetes. The incidence of diabetic cardiovascular disease in patients with diabetes is 2–4 times that in patients without diabetes [1,2], and the fatality rate of acute myocardial infarction is 2–8 times higher in patients with diabetes when compared to their counterparts without diabetes [3]. In DCM, chronic cardiac inflammation is characterized by the continuous loss of cardiomyocytes, which leads to impaired cardiac systolic function [4,5]; however, the exact damage mechanism is yet to be elucidated.

Pyroptosis is a key factor in DCM myocardial inflammation [6,7], and it involves the necrotization and programmed death of inflammatory cells through a mechanism that differs from common cell necrosis and apoptosis pathways [8]. In DCM, hyperglycemia stimulates the activation of inflammasomes in nucleotide-binding oligomerization domain-like receptor protein 3 (NLRP3) [9,10]. This promotes the recruitment of cysteine aspartase-1 (caspase-1) into inflammasomes and its subsequent activation through self-processing [11]. Activated caspase-1 acts on interleukin (IL)-1β, IL-18, and the pore-forming protein gasdermin D (GSDMD), which eventually leads to strong cell lysis and rapid release of pro-inflammatory cytokines [12]. As the core of the signaling cascade, NLRP3 and caspase-1 are considered to be inflammatory markers and drug targets of DCM [13].

Circular RNAs (circRNAs) are endogenous, non-coding RNAs (ncRNAs) that connect through their 3’ and 5’ ends to form a complete loop structure through exon or intron circularization. Therefore, the structures are more stable and more conservative than those of linear RNA and can exist in large, widely distributed quantities in organisms. The circRNA content of certain tissues can be nearly 10 times higher than that of linear RNA. In recent years, an increasing number of researchers have discovered that circRNAs play an important role in gene expression and regulation [14–17]. This occurs mainly through the sponge adsorption of microRNA (miRNA), which antagonizes miRNA inhibition of its downstream target genes, and then regulates the corresponding target genes after transcription [18]. miRNAs are closely related to various diseases, which suggests that circRNAs may also play a regulatory role in disease occurrence and development. It has been reported that CDR1as is expressed on pancreatic islet cells and hinders the function of miR-7, which in turn increases insulin secretion. This observation suggests that CDR1as can affect insulin secretion and β-cell turnover in patients with diabetes [19]. Heart-related circRNA (HRCR) is an endogenous miRNA sponge that regulates the expression of miR-223 and its downstream gene apoptosis repressor with caspase recruitment domain (CARD) (ARC), which is involved in the occurrence of myocardial hypertrophy and heart failure [20]. However, circRNA and the pathogenesis and possible mechanisms of DCM have not yet been reported. According to research of Guo Q et al., circ_0071269 is a newfound circRNA that may be a key regulator involved in hypertrophic cardiomyopathy pathogenesis [21].

miRNAs are short non-coding RNAs that downregulate proteins by binding to a complementary sequence in the 3’-untranslated region (3’-UTR) of the target mRNA [22]. miRNAs have also been shown to regulate and participate in cell pyroptosis [23,24]: miR-148a can promote pyroptosis in alcoholic liver disease; miR-30d directly targets FoxO3a to regulate cardiomyocyte pyroptosis in patients with DCM. However, the role of miR-145 in patients with DCM remains largely unknown.

In this study, we investigated the molecular mechanisms of circ_0071269 and miR-145 in DCM. Our results indicate that circRNAs and miRNAs are potential targets for the treatment of DCM.

## Materials and methods

### Cell culture and transfection

The H9c2 rat embryonic cardiomyocyte cell line was purchased from the Shanghai Cell Bank of the Chinese Academy of Sciences and subcultured with different concentration of glucose: 5.5 mmol/L glucose (Control) and 30 mmol/L glucose (HG) for 24 h at 37°C and 5% CO_2_ conditions. We selected well-growing cells from passages 5–8 for subsequent experiments. Small interfering RNA targeting circ_0071269 (si-circ_0071269) and their respective controls were synthesized by GenePharma (Shanghai, China). miR-145 mimic, miR-control, miR-145 inhibitor, and anti-miR-control were synthesized by RiboBio. The overexpression vector of gasdermin A (GSDMA) was obtained by cloning the GSDMA sequence into a plasmid cloning DNA (pcDNA) vector (RiboBio, Guangzhou, China). Lipofectamine™ 2000 reagent (Invitrogen, Carlsbad, CA, USA) was used to transfect H9c2 cells [25].

#### Animal experiments

Male C57BL/6 mice (18–20 g) were obtained from the animal experiment center of the First Affiliated Hospital of Zhengzhou University. The mice were randomly divided into four groups of ten. Intraperitoneal injection of 50 mg/kg streptozocin (STZ; Sigma-Aldrich, St. Louis, MO, USA) for 5 days was performed to induce diabetes in mice. After 72 h, we detected the caudal vein blood glucose of the model mice by a Contour glucose meter (Roche, Germany), and the diabetic models (DM) were deemed to be success if the blood glucose exceeded 16.7 mmol/L. DM mice were administered with either the circ_0071269 lentivirus-shRNA (DM + sh-circ_0071269) or negative shRNA control (DM + sh-nc) (GenePharma, Shanghai, China). Then, 1 × 10^9^ TU lentivirus-shRNA was dissolved in 50 μL saline and injected into the tail vein of DM mice [7].

### Cell counting Kit-8 (CCK-8) assay

H9c2 cells were cultured in a 96-well plate. When the cell fusion reached the standard level, the cells were treated with drugs and fully washed with phosphate-buffered saline (PBS). Subsequently, 100 μL of CCK-8 solution diluted in DMEM was added (the volume ratio of DMEM and CCK-8 stock solution was 9:1) [26]. After incubating for 2 hours in the cell incubator, the absorbance at 450 nm was measured using a microplate reader.

### *Actinomycin D and RNase R treatment* [27]

To block transcription, 2 mg/mL actinomycin D was added to the cell culture medium, and dimethyl sulfoxide (Sigma-Aldrich, St. Louis, MO, USA) was used as a negative control. For RNase R treatment, 3 U/μg RNase R (Epicenter Technologies, Madison, WI, USA) was added to total RNA (2 μg) and incubated at 37°C for 1 hour. After treatment with actinomycin D and RNase R, cells were subjected to real-time quantitative PCR (qRT-PCR).

### Dual luciferase reporter gene detection

H9c2 cells (5 × 10^3^) were seeded into 96-well plates and cultured until they reached 70% confluence. Subsequently, the luciferase reporter plasmid was transfected into H9c2 cells using Lipofectamine 2000 reagent [28]. After 48 hours, luciferase activity was detected using a dual luciferase reporter gene detection kit (Promega Corporation, Madison, WI, USA).

### Enzyme-linked immunosorbent assay (ELISA), lactate dehydrogenase (LDH) kit

IL-1β and IL-18 concentraion in H9c2 cells of each group was measured by an IL-1β and IL-18 ELISA kit (PI301, PI553; Beyotime, Shanghai, China) following the manufacturers instructions, respectively. Likewise, secrtion of LDH was determined by a LDH test kit (C0016; Beyotime, Shanghai, China) according to the kit instructions [29].

### qRT-PCR

Total RNA was extracted according to the instructions of the RNA extraction kit (Invitrogen, Carlsbad, CA, USA) [30], reverse transcription was carried out using the mRNA reverse transcription kit (High-Capacity cDNA Reverse Transcription Kit; Life Technologies, Grand Island, NY, USA), β-actin was used as the internal reference, and complementary DNA (cDNA) amplification was carried out using the fluorescence quantitative PCR kit. The data were analyzed and quantified using the Ct comparison method (2^−ΔΔCt^). Primers for the PCR reactions were listed as follows: circ_0071269 (Forward, 5’-CCACTGGGCTTGTACCATGT-3’; Reverse, 5’-TCTCCTCGCCTCCAGTTAGT-3’); miR-145 (Forward, 5’-CTCAACTGGTGTCGTGGAGTCGGCAATTCAGTTGAGGAATCCCT-3’; Reverse, 5’-ACACTCCAGCTGGGGTCCAGTTTTCCCAGGAAT-3’); GSDMA (Forward, 5’-TACGTCCGCACCGACTACA-3’; Reverse, 5’-CAGAGTGCTGTTCTGCGAGA-3’); U6 (Forward, 5’-GCUUCGGCAGCACAUAUACUAAAAU-3’; Reverse, 5’-CGCUUCACGAAUUUGCGUGUCAU-3’), β-actin (Forward, 5’-CAGTCGGTTGGATGGAGCAT-3’; Reverse, 5’-AGGCAGGGACTTCCTGTAAC-3’); IL-1β (Forward, 5’-CCACCTCCAGGGACAGGATA-3’; Reverse, 5’-GGGTTCACCGCACTTGAAAC-3’), and IL-18 (Forward, 5’-GGCAGCACTACCAGTTGGAT-3’; Reverse, 5’-AAGGGGTCAGGGGGTATCTC-3’).

### Western blotting

Total protein was extracted according to the manufacturer’s instructions, and the protein concentration was determined by the bicinchoninic acid (BCA) method [30]. After thoroughly mixing the extracted total protein with 5x protein loading buffer at a ratio of 4:1, the protein was denatured at 99°C for 10 minutes. The protein was loaded onto a sodium dodecyl sulfate–polyacrylamide (SDS-PAGE) gel in equal amounts, and the layered gel was run at 55 V for 55 minutes, after which the samples were allowed to reach the bottom of the gel at 100 V. The proteins were then transferred to a polyvinylidene fluoride (PVDF) membrane and blocked with a blocking solution at room temperature for 1 hour. The primary antibodies against NLRP3 and caspase-1 were added, and the antibody-treated membranes were incubated overnight at 4°C. Primary antibodies were as follows: anti-pro-caspase-1 + anti-cleaved-caspase-1 (ab179515, dilution 1:800, Abcam). The samples were then washed three times in Tris-buffered saline with 0.1% Tween® 20 detergent (TBST) buffer, then incubated with goat anti-rabbit secondary antibody at room temperature for 1 hour and finally exposed and developed for analysis. β-Actin was used as an internal reference.

### Flow cytometry

After the cells were trypsinized and collected at 5 × 10^4^ cells/mL, 500 μL Annexin V buffer was added to resuspend the cells, and the cells were transferred to an Eppendorf® (EP) tube. The cells were incubated with 5 μL Annexin V-FITC and 5 μL propidium iodide (PI) for 30 minutes in the dark at room temperature, and cell death was detected by flow cytometry [30].

### Echocardiography

A Vevo1100 high-resolution imaging system (VisualSonics, Toronto, ON, Canada) was applied to evaluate the cardiac function. Mice were anesthetized by injection of 4% chloral hydrate at 30 mg/kg for 30 min [31], and fixed onto a flat plate. The left ventricular ejection fraction (EF) and fractional shortening (FS) were derived.

### Hematoxylin and Eosin (HE) staining

The HE staining was performed as previously reported [32]. Briefly, the cardiac tissues from the left ventricle were fixed by 4% formaldehyde at 4°C for 7 days, and then embedded in paraffin, cut into 5 µm sections. HE staining was performed for histological observation.

### Immunohistochemical (IHC) staining

IHC staining was performed to evaluate the expression of GSDMA in the cardiac tissues from the left ventricle. The tissue sections were treated with 3% hydrogen peroxide, and blocked in 10% goat serum (Sigma-Aldrich, St. Louis, MO, USA) for 1 h. Subsequently, an anti-GSDMA antibody (1:4000; Abcam; ab230768) was incubated with the sections at 4°C overnight, with nonimmune serum as a control. Next, the tissue sections were incubated with a biotin-labeled secondary antibody (1:100; Proteintech; SA00004-6), and the signal was amplified and visualized with the chromogen diaminobenzidine, followed by hematoxylin counterstaining [33].

### Statistical analysis

SPSS19.0 statistical software was utilized for data analysis. Normally distributed measurement data were expressed as mean ± standard error of mean (SEM), an independent sample t-test was used to compare means between two groups, and single factor analysis of variance was used to compare means between multiple groups. Differences were considered statistically significant at *P* < 0.05.

## Results

### The expression of circ_0071269 in H9c2 cells and circRNA characterization

**Figure 1.**
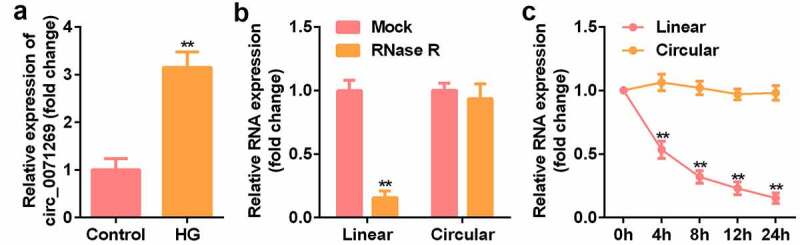
The expression of circ_0071269 and circRNA characterization in H9c2 cells. a: qRT-PCR was used to detect circ_0071269 expression in H9c2 cells treated with high glucose. b: After RNase R treatment, the expression levels of linear-circ_0071269 and circular-circ_0071269 in H9c2 cells were measured by qRT-PCR. c: After treatment with actinomycin D in H9c2 cells at the specified time point, the relative RNA levels were determined by qRT-PCR. All experimental results were obtained by repeating three times. *P < 0.05, **P < 0.01, ***P < 0.001.

To determine the key circRNA that regulates the development of DCM, qRT-PCR verified that the expression level of circ_0071269 in H9c2 cells treated with high glucose was significantly upregulated (Figure 1(a)). The stability and localization of circ_0071269 in H9c2 cells is shown in the figure. Under RNase R treatment, the level of linear-circ_0071269 mRNA significantly reduced, while circular-circ_0071269 was more resistant to RNase R digestion (Figure 1(b)). Quantitative RNA stability determination using actinomycin D and qRT-PCR showed that the half-life of linear-circ_0071269 transcript was approximately 4 hours in H9c2 cells, while the half-life of circular-circ_0071269 mRNA exceeded 24 hours (Figure 1(c)).

### Circ_0071269 suppressed the pyroptosis of H9c2 cells induced by high glucose

**Figure 2.**
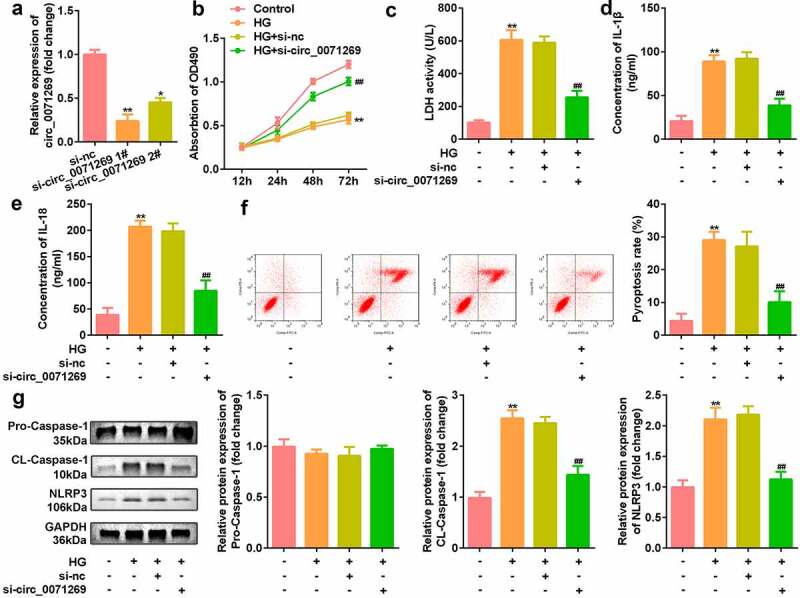
Circ_0071269 knockdown suppresses the pyroptosis of H9c2 cells. a: Two different siRNAs targeting circ_0071269 were detected by qRT-PCR to evaluate the efficiency of circ_0071269 knockdown in H9c2 cells. b: The viability of H9c2 cells treated with high glucose after circ_0071269 knockdown was detected by CCK-8. c: The LDH content of H9c2 cells treated with high glucose after circ_0071269 knockdown was detected using an LDH kit. d-e: The levels of IL-1β and IL-18 in H9c2 cells treated with high glucose after circ_0071269 knockdown were determined using an ELISA kit. f: The apoptosis rate of H9c2 cells treated with high glucose after circ_0071269 knockdown was detected by flow cytometry. G: The protein expression levels of NLRP3 and caspase-1 in H9c2 cells treated with high glucose after circ_0071269 knockdown were detected by Western blotting. All experimental results were obtained by repeating three times. *P < 0.05, **P < 0.01, ***P < 0.001; ^#^*P* < 0.05, ^##^*P* < 0.01, ^###^*P* < 0.001.

Then, effects of inhibited circ_0071269 on cellular functions of H9c2 cells were investigated. As shown in Figure 2(a), si-circ_0071269 significantly decreased the expression of circ_0071269, suggesting successful transfection efficiency (Figure 2(a)). The CCK-8 assay showed that H9c2 cell viability decreased after high glucose treatment, which was antagonized by circ_0071269 knockdown (Figure 2(b)). circ_0071269 knockdown also alleviated the increase in LDH levels induced by high glucose (Figure 2(c)). The ELISA test results showed that the levels of IL-1β and IL-18 in H9c2 cells increased after high glucose treatment and decreased after circ_0071269 knockdown (Figure 2(d,e)). The results of flow cytometry showed that the pyroptosis rate of H9c2 cells increased after high glucose treatment, and this was abrogated by circ_0071269 knockdown (Figure 2(f)). Moreover, the protein expression levels of NLRP3 and cleaved caspase-1 increased in H9c2 cells treated with high glucose and decreased after circ_0071269 knockdown (Figure 2(g)).

### Circ_0071269 acts as a sponge for miR-145

**Figure 3.**
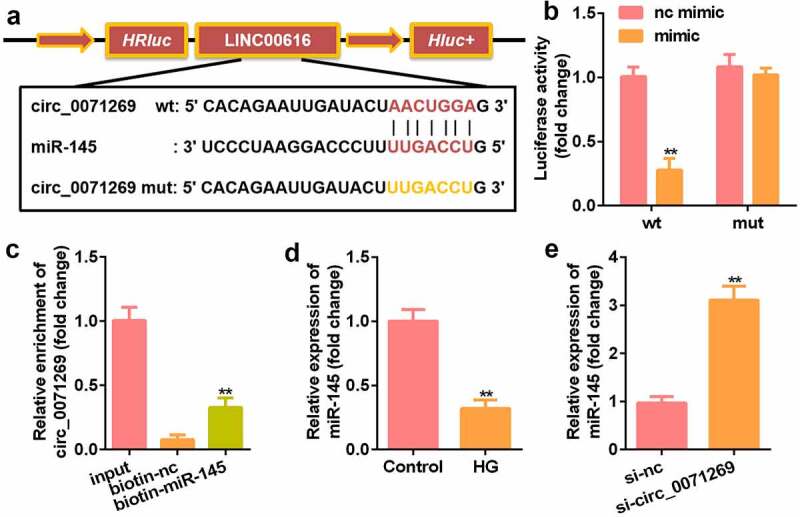
Circ_0071269 acts as a sponge for miR-145. a: Bioinformatics predictions show the targeting relationship between circ_0071269 and miR-145. b: Luciferase activity experiments were performed to verify the targeting relationship between circ_0071269 and miR-145. c: Relative mRNA enrichment of circ_0071269 was captured using biotin-miR-145. d: miR-145 expression reduced in H9c2 cells treated with high glucose. E: miR-145 was elevated in H9c2 cells induced by high glucose that knocked down circ_0071269. All experimental results were obtained by repeating three times. *P < 0.05, **P < 0.01, ***P < 0.001.

We further explored the downstream target genes of circ_0071269 through StarBase v.2.0 and found a binding site between circ_0071269 and miR-145 (Figure 3(a)). The luciferase reporter assay showed that miR-145 mimics reduced the relative luciferase activity of the circ_0071269-WT group (Figure 3(b)). In addition, compared with the biotin-NC group, the amount of circ_0071269 mRNA captured by biotin-145 was significantly higher (Figure 3(c)). The expression of miR-145 significantly decreased in H9c2 cells upon treatment with high glucose (Figure 3(d)), and the expression of miR-145 in H9c2 cells was induced by high glucose circ_0071269 knockdown (Figure 3(e)).

### miR-145 restores the inhibitory effect of circ_0071269 on the progression of H9c2 cells induced by high glucose

**Figure 4.**
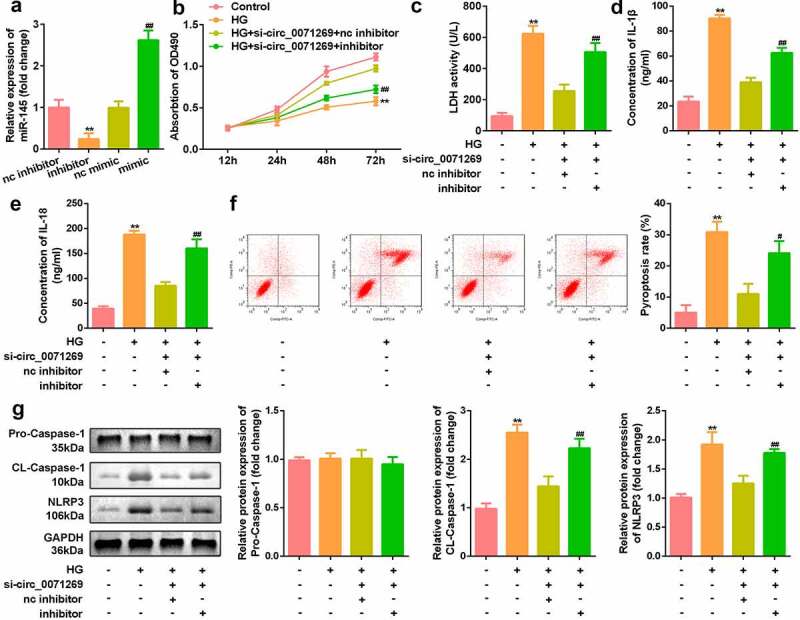
miR-145 restores the inhibitory effect of circ_0071269 on the progression of H9c2. a: The level of miR-145 in H9c2 cells treated with miR-145 inhibitor and miR-145 mimic was measured by qRT-PCR. b: Cell viability detected by CCK-8 assay was suppressed in glucose-stimulated H9c2 cells co-transfected with miR-145 inhibitor and si-circ_0071269. c: The LDH content of H9c2 cells induced by high glucose, which inhibited miR-145 and knocked down circ_0071269, was detected using an LDH kit. d-e: Detection of IL-1β and IL-18 content in H9c2 cells induced by high glucose that inhibited miR-145 and knocked down circ_0071269 were determined using an ELISA kit. F: Flow cytometry detection inhibited miR-145 and knocked down circ_0071269’s high glucose-induced apoptosis rate in H9c2 cells. g: The protein expression levels of NLRP3 and caspase-1 in H9c2 cells induced by high glucose, which inhibited miR-145, while knocking down circ_0071269, were detected by Western blotting. All experimental results were obtained by repeating three times. *P < 0.05, **P < 0.01, ***P < 0.001; ^#^*P* < 0.05, ^##^*P* < 0.01, ^###^*P* < 0.001.

To investigate whether circ_0071269 works through sponging miR-145, we conducted a rescue experiment. H9c2 cells were treated with miR-145 inhibitor and miR-145 mimic to decrease and increase the expression of miR-145, respectively (Figure 4(a)). Notably, the inhibition of miR-145 reversed the effects of circ_0071269 knockdown on the proliferation of H9c2 cells (Figure 4(b)). Moreover, downregulated miR-145 also increased the levels of LDH (Figure 4(c)), IL-1β (Figure 4(d)), IL-18 (Figure 4(e)), pyroptosis rate (Figure 4(f)), and protein expression levels of NLRP3 and cleaved caspase-1 (Figure 4(g)) in H9c2 cells.

### GSDMA is the direct target of miR-145

**Figure 5.**
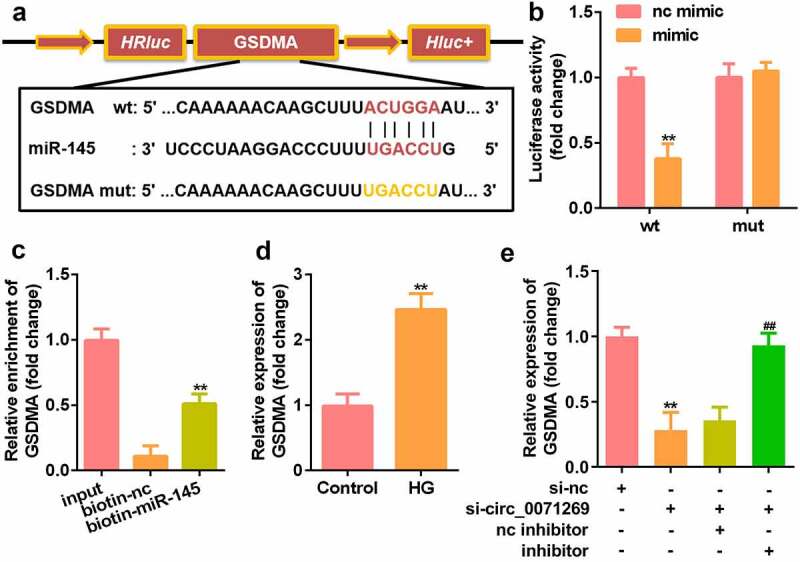
GSDMA is the direct target of miR-145 in H9c2 cells. a: Online prediction software was used to identify the targeting relationship between GSDMA and miR-145. b: Luciferase activity experiments was performed to verify the targeting relationship between GSDMA and miR-145. c: Relative mRNA enrichment of GSDMA was captured using biotin-miR-145. d: GSDMA levels elevated in H9c2 cells treated with high glucose. E: GSDMA increased and decreased in H9c2 cells treated with miR-145 inhibitor and si-circ_0071269, respectively. All experimental results were obtained by repeating three times. *P< 0.05, **P< 0.01, ***P< 0.001.

We searched for the downstream target genes of miR-145 using StarBase v.2.0 and found a binding site between miR-145 and GSDMA (Figure 5(a)). Luciferase reporter gene detection showed that miR-145 mimics reduced the relative luciferase activity of the GSDMA-WT group (Figure 5(b)). In addition, compared with the biotin-NC group, the GSDMA mRNA captured by biotin-145 was significantly higher (Figure 5(c)). qRT-PCR also detected a significant increase in GSDMA expression in H9c2 cells treated with high glucose (Figure 5(d)). The downregulation of GSDMA induced by si-circ_0071269 was alleviated by the miR-145 inhibitor (Figure 5(e)).

### Overexpression of GSDMA reversed the effects of miR-145 in H9c2 cells

**Figure 6.**
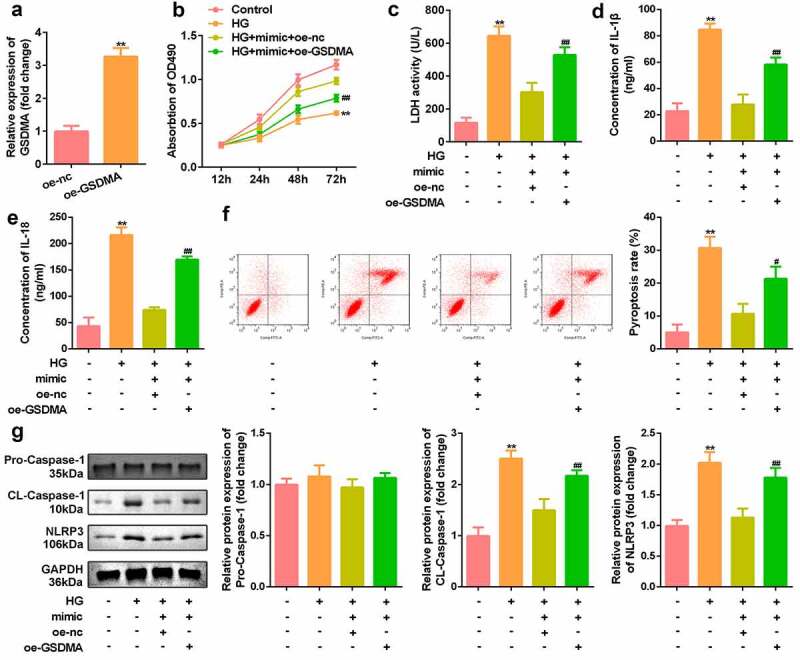
Overexpression of GSDMA reversed the effect of miR-145 on H9c2. a: The level of GSDMA in H9c2 cells overexpressing GSDMA was measured by qRT-PCR. b: Cell viability of miR-145 mimic transfected simultaneously with overexpressing GSDMA was detected by CCK-8. c: The LDH content of cells overexpressing GSDMA upon transfection of the miR-145 mimic was determined using the LDH kit. D-E: The levels of IL-1β and IL-18 in cells overexpressing GSDMA upon transfection of the miR-145 mimic were determined using an ELISA kit. F: Flow cytometry was used to detect the high apoptotic rate associated with GSDMA overexpression in cells transfected with the miR-145 mimic. g: Western blotting was used to detect the protein expression levels of NLRP3 and caspase-1 in cells transfected with miR-145 mimic and overexpressing GSDMA. All experimental results were obtained by repeating three times. *P < 0.05, **P < 0.01, ***P < 0.001; ^#^*P* < 0.05, ^##^*P* < 0.01, ^###^*P* < 0.001.

Our rescue experiment results showed that GSDMA was upregulated by GSDMA overexpression plasmids (Figure 6(a)). At the same time, overexpression of GSDMA reversed the effects of the miR-145 mimic on H9c2 cell viability (Figure 6(b)). Moreover, GSDMA overexpression suppressed the effects of miR-145 overexpression on LDH (Figure 6(c)), IL-1β (Figure 6(d)), and IL-18 contents (Figure 6(e)), pyroptosis rate (Figure 6(f)), and the protein expression levels of NLRP3 and cleaved caspase-1 (Figure 6(g)).

### Knockdown of circ_0071269 attenuated cardiac dysfunction of DM mice

**Figure 7.**
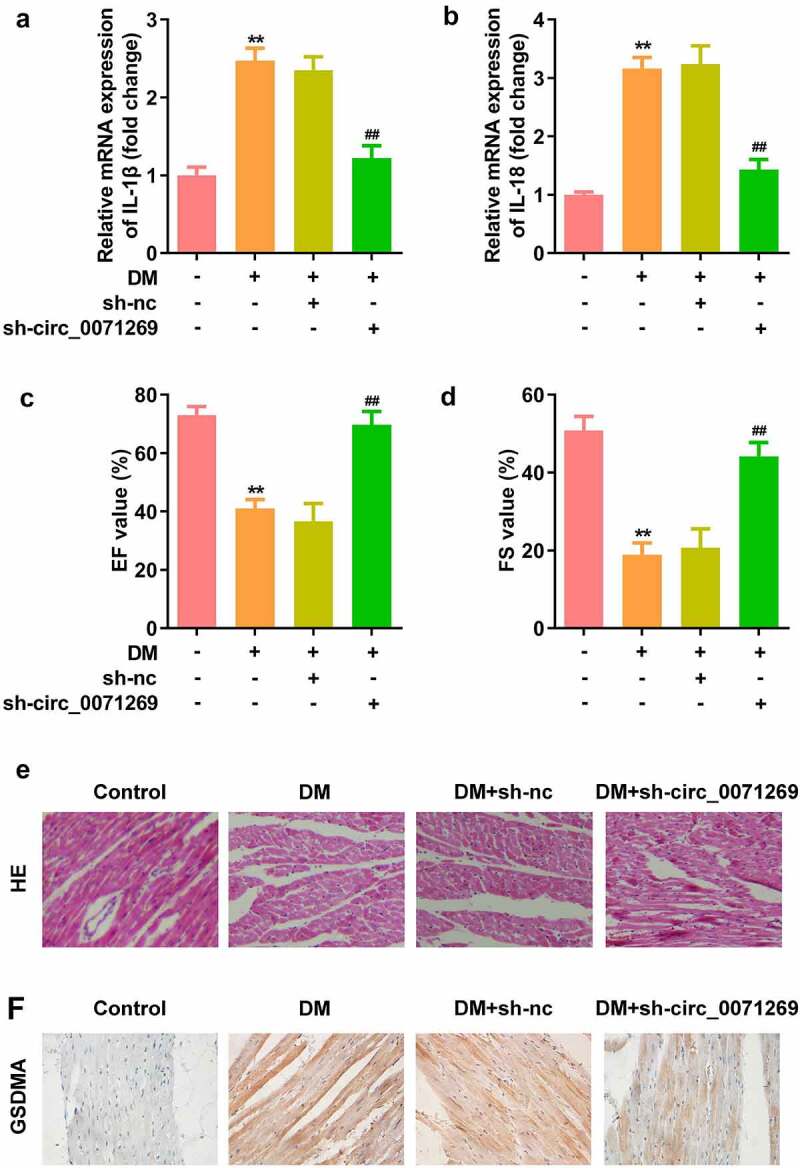
Knockdown of circ_0071269 attenuated cardiac dysfunction of DM mice. a-b: mRNA expression levels of IL-1β and IL-18 in cardiac tissue of the DM mice determined by qRT-PCR. c-d: The echocardiographic data including EF and FS were determined by Echocardiography. e: Images of cardiac tissue stained by HE staining assay. f: The expression of GSDMA obtained from IHC method. All experimental results were obtained by repeating three times. **P < 0.01; ^##^*P* < 0.01.

As indicated in Figure 7(a,b), mRNA expression levels of IL-1β and IL-18 in cardiac tissue of the DM mice were decreased by inhibition of circ_0071269. The echocardiographic data including EF and FS were decreased in the DM group, and were restored by knockdown of circ_0071269 (Figure 7(c,d)). Morphologically, HE staining revealed that obvious hypertrophy and necrosis were evident in the myocardial cells of the DM mice group, and the morphology of myocardial cells in DM + sh-circ_0071269 group recovered, intercellular spaces were reduced, and the arrangements were regular (Figure 7(e)). Furthermore, higher protein level of GSDMA was also found in DM mice group compared with normal group by IHC staining, while inhibition of circ_0071269 decreased the protein level of GSDMA.

## Discussion

In this study, we identified an altered circRNA associated with DCM and further clarified the different cellular pathways regulated by this circRNA. Specifically, circ_0071269 directly targeted miR-145 and regulated the proliferation and pyroptosis of H9c2 cells by upregulating GSDMA.

circRNA is a widely studied type of ncRNA that is highly conserved in mammals. Recent studies revealed that in DCM, circ_000203 exacerbated myocardial fibrosis by increasing Col1a2 and connective tissue growth factor (CTGF) via miR-26b-5p [34]. circRNA_010567 also promotes myocardial fibrosis via the miR-141/TGF-β1 pathway in DCM [35]. Salzman et al. [36] used RNA sequencing (RNA-Seq) to confirm for the first time that circRNAs are widespread in human somatic cells. Subsequent studies have shown that circRNA is abundantly expressed in mammalian cells [37,38], and its sequence is evolutionarily conserved [14]. This study confirmed that circ_0071269 is highly expressed in H9c2 cells induced by high glucose.

Pyroptosis is a type of programmed cell death related to the activation of inflammation [39]. It has been reported that hyperglycemia activates NLRP3 inflammasome, thus promoting pro-caspase-1 to caspase-1. The cleaved caspase-1 is then capable of converting the pro-interleukin-1β (pro-IL-1β) and pro-interleukin-18 (pro-IL-18) into matured IL-1β/IL-18 [9]. Our data revealed that inhibition of circ_0071269 significantly improved cell viability, LDH content, IL-1β and IL-18 content, apoptosis rate, and protein levels of NLRP3 and caspase-1, which demonstrated that pyroptosis of DCM model cells were suppressed by konckdown of circ_0071269. We are the first investigators to clarify the differential expression of circ_0071269 in the DCM model.

As a sponge of miRNA, circRNA is most widely studied in the cytoplasm [40,41]. Several circRNA-miRNA interactions have been shown to be essential for the occurrence and progression of DCM. Prior studies have found that the expression of a large number of miRNAs in DCM is abnormal. Recent studies have confirmed that miR-145 can protect against rat myocardial infarction by targeting PDCD4 [42]. Prior studies have also shown that miR-145 can directly target PDCD4 to protect human cardiomyocytes from high glucose (HG)-induced apoptosis [43]. In this study, according to our bioinformatics data, there was a binding site for miR-145 on circ_0071269. We then performed a function gain experiment and established a regulatory relationship between circ_0071269 and miR-145. We found that miR-145 was significantly downregulated in H9c2 cells induced by high glucose. In particular, *in vitro* rescue experiments revealed that miR-145 recovered the inhibitory effect of circ_0071269 on the progression of H9c2 cells induced by high glucose.

Pyroptosis is also known as gasdermin (GSDM)-mediated programmed cell death, and the GSDM protein family mediates cell pyroptosis. Gasdermin is a protein involved in cell pyrolysis and plays an important role in the pathogenesis of stroke. These proteins contain a novel membrane pore formation domain [44]. Saeki et al. [45] found in related studies on tumor cells that GSDMA is a tumor suppressor gene, and transforming growth factor (TGF)-β can induce cell death by upregulating GSDMA through the transcription factor LMO1. Thus, GSDMA plays an important role in inducing cell death and inflammation and is a key mediator of pyroptosis [46,47]. Yue et al. [48] found that anthocyanins can promote activation of caspase-1 to cleave GSDMD, induce pyroptosis in oral squamous cell carcinoma, and inhibit tumor progression. In non-small cell lung cancer, GSDMD can be cleaved by caspases, which causes cancer cells to scorch [49]. Pizato et al. [50] found that treating breast cancer cells with docosahexaenoic acid (DHA) can activate caspase-1 to cleave GSDMD and induce pyroptosis in breast cancer cells. However, the mechanism of GSDMA in pyroptosis has remained unclear for a long time. In our study, we found that GSDMA expression was significantly reduced in H9c2 cells induced by high glucose. In addition, overexpression of GSDMA reversed the effect of miR-145 in H9c2 cells induced by high glucose. These results indicate that miR-145 directly targets GSDMA to regulate pyroptosis in H9c2 cells. Moreover, circ_0071269 functions as a sponge of miR-145 to upregulate GSDMA, which plays a key role in cell pyroptosis. Furthermore, results of *in vivo* experiment also illustrated that inhibition of circ_0071269 helped to attenuate cardiac dysfunction of DM mice. These findings suggest that circ_0071269 participates in the development of DCM by regulating the miR-145/GSDMA axis.

## Conclusion

In summary, a high level of circ_0071269 exacerbated the development of DCM. circ_0071269 promotes pyroptosis of cardiomyocytes via the miR-145/GSDMA axis. Therefore, the circ_0071269/miR-145/GSDMA axis is a potential candidate for molecular targeted therapeutic intervention for DCM.

## Data Availability

The datasets used and analyzed during the current study are available from the corresponding author on reasonable request.

## Research highlights

- circ_0071269 was aberrant upregulated in H9c2 cells upon treatment with high glucose.
- Kncokdown of circ_0071269 inhibited the inflammatory response, cytotoxicity, and pyroptosis of H9c2 cells *in vitro*, and attenuated cardiac dysfunction of DM mice *in vivo*.
- circ_0071269 may promote the development of DCM through the miR-145/GSDMA axis.
